# The Use of Imaging Techniques in the Diagnosis of Dermatoses of the Scalp

**DOI:** 10.3390/medicina61091553

**Published:** 2025-08-29

**Authors:** Aleksandra Kuźniak-Jodłowska, Magdalena Jałowska, Grzegorz Nowaczyk, Aleksandra Dańczak-Pazdrowska

**Affiliations:** 1 Department of Dermatology, Poznan University of Medical Sciences, 60-355 Poznan, Poland; mjalowska@ump.edu.pl (M.J.); aleksandra.danczak-pazdrowska@ump.edu.pl (A.D.-P.); 2 Doctoral School, Poznan University of Medical Sciences, 60-812 Poznan, Poland; 3 NanoBioMedical Center, Adam Mickiewicz University, 61-614 Poznan, Poland; grzegorz.nowaczyk@amu.edu.pl

**Keywords:** non-invasive techniques, scalp dermatoses diagnosis, biomedical research, trichoscopy, optical coherence tomography, phototrichogram, TrichoScan, microscopy, ultrasonography, trichogram

## Abstract

Scalp diseases are a common issue affecting patients’ self-esteem and quality of life. Currently, trichoscopy is the foundation of diagnostics; however, it does not always provide sufficient sensitivity and specificity. In uncertain cases, scalp biopsy remains the gold standard, though it is an invasive method and not well accepted by patients. In recent years, new non-invasive diagnostic methods have been developed and modernized. This article discusses imaging techniques, emphasizing their development over time as well as their advantages and limitations in the diagnosis of scalp dermatoses.

## 1. Introduction

Historically, hair has been an object of study and observation in various civilizations. Ancient societies, such as the Egyptians and Greeks, recognized the importance of hair health as a reflection of overall vitality, linking hair loss or changes in hair quality to potential illnesses. Hippocrates was the first to use the term “alopecia” to describe a patient in whom he observed hair loss [1]. Early investigations focused on the anatomical and physiological characteristics of hair, gradually advancing to a deeper understanding of hair follicle cycles, scalp dermatoses, and their clinical implications.

Scalp dermatoses, such as psoriasis, seborrheic dermatitis, lichen planus, discoid lupus erythematosus, or contact dermatitis, may present with an ambiguous clinical picture, and in some cases, a diagnosis based solely on clinical findings is not possible. On the other hand, a precise and reliable diagnosis of scalp dermatoses is essential for developing an effective therapeutic plan. It is worth noting that recent years have seen tremendous progress in the diagnosis of alopecia, with a continued dynamic development of new research techniques. Numerous methods are available for diagnosing scalp diseases. Hair assessment methods can be classified into three main categories based on their level of invasiveness: non-invasive techniques (e.g., trichoscopy, phototrichogram, TrichoScan, optical coherence tomography, reflectance confocal microscopy, atomic force microscopy, high-frequency ultrasonography, and scanning or transmission electron microscopy, when applied to cut hair shafts); semi-invasive techniques (e.g., trichogram, light microscopy, and polarized light microscopy), which involve hair plucking but do not require tissue sampling; and invasive techniques (e.g., scalp biopsy, transmission electron microscopy, and ex vivo confocal laser scanning microscopy), all of which require tissue collection and enable direct evaluation of hair follicles [2].

The impact of scalp diseases, whether they involve a normal hair structure and count and/or hair loss, on quality of life is a significant factor driving the development of new non-invasive diagnostic methods.

The aim of this article is to provide an overview of scalp diagnostic techniques, highlighting their advantages and limitations. A summary table at the end consolidates key aspects such as resolution, examination depth, preparation procedures, and diagnostic evaluation capabilities.

## 2. Non-Invasive Techniques

### 2.1. Trichoscopy

In 2006, “trichoscopy” became a well-known term for videodermatoscopy of hair, scalp, eyebrows, and eyelashes [3]. It is a diagnostic method based on dermoscopy/videodermoscopy technique. A trichoscopic examination can be performed using a handheld dermoscope (at 10× magnification) or a videodermoscope (with magnifications ranging from 20× to 1000×). The most commonly used magnifications are 20- and 70-fold. This method allows for the observation and evaluation of structures at the epidermal level, the epidermal–dermal junction, and the upper layers of the dermis, as well as the hair, including, particularly, the evaluation of hair follicle openings, which constitute the upper part of the hair canal, the surrounding skin of the follicle, the blood vessels, and the hair shafts. Dermatoscopic features can be classified into follicular, interfollicular, and combined categories. Follicular features most commonly include broken hair, black dots, single hair follicle unit, short vellus hair, upright hair, yellow dots, and white dots, while interfollicular features include honeycomb pigmentation and arborizing red lines, with combined features involving epidermal scale and pustule [4]. A detailed analysis of the practical applications of trichoscopy has highlighted its importance in diagnosing and monitoring common hair and scalp disorders. In 2007, Tosti published the first atlas containing trichoscopy images [5], and later in 2012, Rudnicka and Rakowska created “The Atlas of Trichoscopy,” the pioneering book that organized and systematized scientific knowledge on trichoscopy [6]. The examination in trichoscopy can be performed using polarized or non-polarized light, either with immersion medium (placed between the instrument and the skin) or through dry dermoscopy. The immersion medium used in trichoscopy may be water, ultrasound gel, aqueous gel, liquid paraffin, alcohol, or oil. The choice of a specific immersion fluid depends on individual preferences [7]. Without immersion, the focus is on observing dry scale or perifollicular exfoliation, which is visible in its natural state and can indicate conditions like dandruff or psoriasis. With immersion, the examination provides enhanced visualization of deeper structures, such as hair shafts, follicular openings, and blood vessels. Immersion fluids, especially oil-based ones like liquid paraffin, improve contrast and clarity, aiding in the detection of subtle details like sebaceous gland activity or pigmentation. Trichoscopy with UV light (365 nm) provides new opportunities for diagnosing alopecia. UV-enhanced trichoscopy (UVET) uses UV-A light to induce characteristic fluorescence in the presence of certain skin conditions. For example, Cutibacterium acnes, found in acne comedones, emits a red-orange fluorescence due to porphyrin production when exposed to UV light. Compacted keratin typically produces a whitish-yellow fluorescence. The “Starry Night Sky Sign” may help predict treatment efficacy in conditions like frontal fibrosing alopecia. Positive fluorescence indicates the presence of follicular Cutibacterium acnes, suggesting potential preservation of follicular unit viability and a more favorable prognosis. In contrast, fibrosed follicular units do not show this fluorescence [8]. Additionally, UV trichoscopy facilitates the differentiation between cicatricial and non-cicatricial hair loss while also helping to distinguish between psoriasis and seborrheic dermatitis [9,10]. In some cases, additional examination of eyebrow or eyelash hairs and other body hair can provide diagnostically relevant information.

To sum up, one of the most significant advances brought by trichoscopy is the ability to assess the structure of the hair shaft without the need for hair samples. Trichoscopy allows for the examination of the scalp with regard to hairs with abnormal structures and it helps avoid unnecessary biopsies. Moreover, when biopsy is necessary, it assists in choosing the ideal biopsy site. It is also used for measurements and assessments of disease activity, severity, and prognosis, as well as for tailoring therapeutic responses. It can also be combined with photography and digital imaging for clinical documentation purposes [11]. The advantage of trichoscopy is its ability to quickly examine large areas while remaining non-invasive, real time, cost effective, easy to use, and portable, making it a practical tool in clinical settings; however, its diagnostic accuracy may be compromised if the physician misinterprets the significance of the observed structures [7]. The comparative analysis of non-invasive techniques is presented in Table 1.

### 2.2. Optical Coherence Tomography Scalp Imaging

Optical coherence tomography (OCT), first introduced in 1991 by Huang et al. for ophthalmologic imaging, was later adapted for dermatologic applications, including scalp and hair assessment. It is a non-invasive, in vivo imaging technique that employs low-coherence interferometry to provide high-resolution, cross-sectional images of biological tissues, including the scalp and hair follicles. It offers axial resolutions between 3 and 15 μm, with a maximum penetration depth of up to 1–2 mm, allowing for visualization of the epidermis, dermis, hair shafts, and follicular structures without the need for hair trimming or plucking [12]. Unlike trichoscopy, which assesses only the surface structures of the scalp, OCT enables subsurface imaging of deeper components such as hair follicles, sebaceous glands, and dermal blood vessels, enhancing the diagnostic evaluation of various scalp and hair disorders [13]. Moreover, OCT allows for real-time, in vivo imaging and dynamic monitoring of scalp morphology and vascular changes during treatment or disease progression [14]. It is particularly valuable in early-stage cicatricial alopecias, such as frontal fibrosing alopecia, where inflammatory perifollicular changes can be visualized, but is less informative in advanced scarring alopecia with complete follicular destruction (late-stage lichen planopilaris or discoid lupus) and in hair shaft disorders, such as monilethrix or trichorrhexis nodosa, where the pathology is confined to the shaft [12,13,14].

### 2.3. Phototrichogram

The phototrichogram (PTG) is a non-invasive method used to assess hair growth and its life cycle by taking a series of photographs of a shaved area of the scalp. The first image is captured immediately after shaving and the subsequent one about 72 h later. This allows for the measurement of hair length increase and determination of the ratio of hairs in the anagen (growth) phase to those in the telogen (resting) phase. The method was first proposed in the 1970s by Saitoh, as well as Fiquet and Courtois, and later developed and refined by Bouhanna in the 1980s [15]. Thanks to these advancements, the phototrichogram has become a precise tool for studying hair cycles and monitoring treatment outcomes in scalp disorders. The PTG enables the measurement of parameters such as hair density, percentage of telogen hairs, growth rate, hair shaft diameter, and the proportion of fine hairs. The contrast-enhanced version (CE-PTG) improves the detection of thin and lightly pigmented hairs, making it particularly useful in diagnosing androgenetic alopecia. Today, the phototrichogram is a valuable diagnostic tool and a reliable method for monitoring the effectiveness of treatments for scalp hair disorders [16].

### 2.4. TrichoScan

TrichoScan is a computerized version of the phototrichogram, introduced in 2001 as a fully automated method for assessing hair growth parameters [17]. The system evaluates hair density, diameter, the percentage of hairs in the anagen and telogen phases, as well as the growth rate. The procedure involves shaving an area of the scalp (approximately 2 cm^2^), capturing an image at 20- and 70-fold magnifications, followed by computerized image analysis [18]. The detection limit for hair thickness is 5 μm, allowing for the evaluation of even thin and miniaturized hairs [17]. TrichoScan provides objective, quantitative data, making it especially useful for monitoring treatment outcomes. Unlike trichoscopy, which relies on visual and often subjective assessment, TrichoScan offers standardized, reproducible results, although it requires shaving a small area of the scalp. This method is especially valuable for the evaluation of androgenetic alopecia, alopecia areata, and telogen effluvium but has limited utility in advanced scarring alopecias, where follicles are permanently destroyed [11,17].

### 2.5. Transmission Electron Microscopy and Scanning Electron Microscopy

The first transmission electron microscope was built in 1931 by Ernst Ruska from the Institute of Electrical Engineering at the Higher Technical School in Berlin, while the concept of the scanning electron microscope was proposed by German physicist Max Knoll in 1935. In 1955, an electron microscope was used to observe the morphology of wool fibers [33]. To date, the internal structure and chemical composition of hair cuticles have been studied and described using TEM of hair cross-sections. This has revealed that each cuticle cell consists of seven layers with different compositions and properties. TEM is primarily used for studying hair follicles [19]. Hair samples require processing, including embedding in resin, before visualization using TEM [20]. TEM allows for high-resolution imaging, typically between 0.1 and 0.2 nm. Hair shaft analysis for TEM requires only cut hair, which represents a non-invasive sampling method, particularly suitable for cosmetic and structural studies. However, when the hair follicle or surrounding scalp tissue is the target of investigation, a scalp biopsy is necessary, rendering the procedure invasive. Sample preparation for SEM analysis involves vacuum exposure and metal coating, which may alter the hair surface. Scanning electron microscopy (SEM) is ideal for examining the hair shaft, enabling high-resolution three-dimensional reconstruction of surface features, with a resolution range from 2–5 nm to 0.1–0.4 nm, allowing for detailed observation of both the external surface and the internal structure of the hair. It can detect subtle changes caused by cosmetic treatments such as bleaching, coloring, and straightening [34,35]. SEM also allows for the rapid examination of the hair’s chemical composition for the presence of drugs and other substances. Coroaba et al. used SEM and TEM to study the impact of alopecia areata on the structure and composition of human hair. Lima et al. evaluated the effect of hair straighteners on damage to the cuticle and cortex of hair due to heat loss using SEM. They demonstrated that surface damage to hair depends on the analyzed ethnic origin (Caucasian and Asian hair) [36]. TEM enables ultrastructural analysis of the internal architecture of the hair, making it essential for studying follicular pathology and subcellular alterations. In contrast, SEM excels in assessing surface morphology, providing rapid, high-resolution imaging of the external hair shaft without invasive preparation. Compared to trichoscopy, both TEM and SEM offer superior resolution and depth, allowing for a more detailed examination of hair ultrastructure, composition, and morphological changes that trichoscopy cannot visualize. However, these are ex vivo techniques, requiring hair sample collection.

### 2.6. Atomic Force Microscopy

Atomic force microscopy (AFM), as a type of scanning probe microscopy (SPM), provides images with very high horizontal 0.1 nm, vertical 0.01 nm, and atomic-level resolutions while preserving sample integrity in a manner similar to confocal microscopy [37]. In 1986, Binnig, Quate, and Gerber from Stanford University developed the concept of Atomic force microscopy (AFM), introducing a new imaging technique that allows for the study of surfaces with atomic-level resolution [38]. Samples can be observed in air, liquid, or vacuum, making it possible to study the properties of living cells in their natural or near-natural states [21]. To date, AFM research in medicine has focused on cancer, neurodegenerative diseases, osteoporosis, specific DNA fragments, and ligand–receptor interactions [39,40,41,42]. This technique has led to the identification of new surface features of hair that were not visible by SEM (e.g., roughness and the degree of sheath cell detachment from the hair surface) [43]. Hair samples for AFM may be collected either by cutting or plucking, depending on the purpose and specific requirements of the analysis. Researchers have mainly focused on the impact of external factors such as conditioners, shampoos, coloring, and perming on the aforementioned hair features [44,45,46,47]. Additionally, the impact of internal factors such as aging has also been investigated [48]. This is useful when the effect of modification of the sample surface is suspected [43,49]. There is limited research dedicated to scalp dermatoses. An analysis of morphological changes in human hair depending on environmental conditions was carried out in 1995 by S. D. O’Connor [50]. In 2005, LaTorre used AFM to assess that the physical properties of human hair fibers vary significantly depending on ethnic origin [51]. In 2013, Kyung Sook Kim demonstrated differences in hair structure between patients with scalp psoriasis and seborrheic dermatitis using AFM [52]. Shin et al. used AFM to study the morphological properties of hair taken from the scalps of patients with psoriasis. Their work confirmed the generalized nature of psoriasis, as the hair shafts of psoriasis patients showed the same macrodepressions observed in nails [53]. In 2024, researchers from Rzeszów proposed creating a characterization of healthy hair based on AFM images, which could be used by dermatologists to study hair in patients with various hair diseases [22]. AFM can be used to accurately determine hair shaft thickness, which is not possible via electron microscopy [54]. Although AFM provides detailed nanoscale imaging, trichoscopy continues to be favored in clinical settings due to its speed, simplicity, and availability.

### 2.7. Reflectance Confocal Microscopy

Reflectance confocal microscopy (RCM) was first developed in the 1990s by Rajadhyaksha et al. as a tool for quasi-histological imaging, allowing for tissue examination without the need for biopsy [23]. This technique, with a penetration depth of up to approximately 200 µm, enables the visualization of only the superficial parts of hair follicles and hair shafts [55]. RCM has since been widely applied in dermatology, particularly for non-invasive monitoring of skin diseases and hair disorders. In 2008, Rudnicka et al. demonstrated that reflectance confocal laser scanning microscopy (R-CSLM) enables the precise measurement of hair shaft structures, including the thickness of the shaft, medulla, cortex, and cuticle, which are typically assessable in most terminal hairs [24]. The lateral resolution of RCM ranges from 0.5 to 1 μm, while the axial resolution (vertical layer thickness) is 3 to 5 μm, reaching a depth of approximately 150–200 μm, depending on anatomical location [23,25]. In vivo RCM has proven to be a non-invasive and innovative tool for monitoring subclinical treatment progress in patients with androgenetic alopecia and evaluating the effects of cosmetic procedures [21,56]. Additionally, case studies suggest its potential use as a diagnostic tool for alopecia areata, genetic hair disorders, and other hair loss conditions [55]. A strong correlation between RCM and histopathology has been established, reinforcing its role in bridging clinical assessment, trichoscopy, and histopathological analysis. RCM is particularly advantageous due to its ability to provide real-time, cellular-level imaging without the need for tissue excision or staining. As such, it plays an important role in non-invasive trichological diagnostics, especially in treatment monitoring.

### 2.8. High-Frequency Ultrasonography

Ultrasound technology was first introduced to dermatology in 1979 when Alexander and Miller utilized a 15 MHz frequency to assess skin thickness. Since then, new applications of high-frequency ultrasonography (HF-USG) have emerged, providing clinicians with an additional tool in their daily practice [57]. High-frequency ultrasonography (HF-USG), operating at 20–48 MHz, and ultra-high-frequency ultrasound (UHFUS, >48 MHz) are non-invasive imaging tools providing in vivo detailed visualization of the skin, scalp, and hair follicles. Compared to HF-USG, UHFUS offers superior axial resolution, enabling the assessment of hair shafts within follicles before their emergence on the skin surface and allowing for earlier detection of subtle pathological changes [26]. They allow for the real-time assessment of structural changes, making it useful for diagnosing and monitoring dermatological conditions. However, in certain cases, evaluating the hair follicles requires shaving the skin in the area being examined. In alopecia areata (AA), HF-USG typically reveals hypoechoic peri- and intrafollicular areas indicative of inflammation. However, UHFUS enables the identification of three characteristic features of AA: empty follicles, small ovoid follicles, and perifollicular hyperechogenicity in the subcutis [27]. In androgenetic alopecia (AGA), it reveals follicular miniaturization and increased dermal echogenicity. Moreover, in scarring alopecias like lichen planopilaris (LPP), it identifies dermal thickening and follicular destruction, indicating fibrosis [28]. Advances like Doppler imaging may enhance its precision in assessing vascular and inflammatory conditions. HF-USG is a promising tool for dermatology, improving the diagnosis and management of alopecia. Its continued development and integration into clinical practice could significantly enhance patient care.

## 3. Semi-Invasive Techniques

### 3.1. Trichogram

The trichogram is a semi-invasive, rapid, and cost-effective diagnostic technique routinely utilized in dermatology since its initial description in 1964. Despite the development of more advanced imaging methods, it remains a valuable tool, particularly for the evaluation of acute telogen effluvium. The procedure involves the epilation of approximately 50–100 hairs from standardized scalp regions, most commonly the frontal and occipital areas. Hairs are extracted with a single, firm traction using forceps positioned approximately 0.5 cm from the scalp, in alignment with the natural direction of hair growth. To reduce external variables, patients are advised to refrain from washing or applying cosmetic products to the hair for at least five days prior to sampling. Microscopic evaluation focuses on determining the growth phase of each hair, based on the morphology and pigmentation of the hair bulb, the presence of root sheaths, and the angulation of the hair shaft relative to the bulb. This allows for the classification of hairs into the anagen, catagen, and telogen phases, along with identification of dystrophic hairs. Examinations are typically performed using a 4× objective lens, with higher magnifications (10× or 40×) applied when more detailed morphological analysis is necessary. Results are presented as the percentage distribution of hairs in the respective growth phases, providing essential diagnostic information and aiding in the assessment of disease activity and therapeutic response [29].

### 3.2. Light Microscopy

Classical light microscopy has played a pivotal role in the study of hair since its creation in the 17th century. Antonie van Leeuwenhoek, widely celebrated as the father of microscopy, was among the earliest to observe hair through a microscope [30]. By the 19th century, light microscopy became essential in diagnosing hair disorders. This technique employs a series of lenses to magnify the sample by 10 to 1000 times, with a resolution of 0.2 μm depending on the lens type and numerical aperture [21]. Light microscopy enables the visualization of non-specific changes such as weathering and splits in distal shafts, mild flattening, twisting, or grooving. It is particularly useful for genetic dystrophies and many acquired infectious diseases, such as pediculosis and tinea capitis, as well as non-infectious conditions like loose anagen syndrome. Typically, several to dozens of hairs are collected for examination, but in some diseases, finding a single hair with a characteristic anomaly that allows for a proper diagnosis requires collecting a significantly larger number of hairs, such as in the case of suspected Netherton syndrome. Light microscopy offers high magnification for detecting structural hair defects but, in contrast to trichoscopy, requires sample collection and preparation. It enables the detailed examination of hair morphology ex vivo, making it especially useful in the assessment of hair shaft disorders. Additionally, it serves as a standard tool for quantitative evaluation in trichogram analysis.

### 3.3. Polarized Light Microscopy

The polarized light microscope has proven to be an invaluable analytical tool since its first documented use around 1834 [31]. PLM is one of the most popular forensic microscopy techniques, capable of determining the presence of a variety of substances with high precision in a single analysis, such as minerals, vitamins, amino acids, and hormones, e.g., sex hormones [32]. Additionally, it allows for the observation of melanin granules deposited in the hair shaft [58]. The resolution is similar to standard light microscopy (0.2 μm) [59]. It seems that this method can also be useful in dermatological assessments, as it allows for a series of precise measurements of the hair, hair follicles, including an assessment of the percentage of hair in the anagen, catagen, and telogen phases, as well as hair diameter. PLM can be used in the diagnosis of both congenital and acquired abnormalities of the hair shaft, including monilethrix, pili annulati [60], Chediak–Higashi syndrome, and Griscelli syndrome [61]. It is worth noting that the “tiger tail” appearance of hair, a characteristic feature of trichothiodystrophy, which is not visible under standard light microscopy, can be detected using PLM [62]. Polarized light microscopy enhances contrast and allows for the detailed visualization of hair shaft birefringence, aiding in diagnosing structural abnormalities. To sum up, compared to trichoscopy, polarized light microscopy (PLM) is a semi-invasive, ex vivo technique requiring hair plucking rather than cutting, offering the ability to assess internal hair fiber composition and to perform a trichogram.

## 4. Invasive Techniques

### Ex Vivo Confocal Laser Scanning Microscopy (CLSM)

Although RCM is used in vivo, the broader platform of confocal laser scanning microscopy (CLSM) is also employed ex vivo for detailed imaging of hair and scalp structures. Ex vivo CLSM can operate in both reflection and fluorescence modes. The fluorescence technique enhances visualization of internal hair structures, with fluorescent probes outlining the geometry of cortical and medullary cells based on their lipophilic or hydrophilic properties [25]. This method provides a horizontal resolution below 1.25 μm, a vertical resolution under 5 μm, and a maximum imaging depth of 200 μm, depending on the tissue type. With up to 550× magnification and an image resolution of 1024 × 1024 pixels, ex vivo CLSM ensures high-definition imaging without damaging the excised tissue. Following imaging, samples can be formalin-fixed, paraffin-embedded, and stained for standard histopathological evaluation [25]. Ex vivo CLSM, unlike RCM, requires prior tissue excision and is therefore classified as an invasive technique. However, it offers the significant advantage of combining high-resolution optical imaging with subsequent histological processing of the same sample, enabling direct comparison between confocal and classical microscopic findings. This makes CLSM a valuable tool in research settings focused on hair follicles.

## 5. Conclusions

There are numerous dermatoses affecting the scalp that can impact the condition of the hair. Differential diagnosis, especially in cases where trichoscopy is ambiguous, largely relies on histological examination of the skin, which can be a discomforting procedure in this particular area. Innovative techniques aim to identify differences in hair structure associated with various dermatoses. Insight into hair structure may allow for the selection of prognostic markers and potential monitoring of therapeutic strategies. This could enable an expansion of the diagnostic arsenal for scalp dermatoses with innovative non-invasive hair assessment techniques, thereby eliminating the need for invasive procedures to confirm the disease. Looking ahead, the convergence of advanced imaging modalities, high-resolution digital capture, and AI-driven image analysis holds the promise of earlier, more precise detection of scalp dermatoses.

A summary of the information regarding the techniques is presented in Table 1.

## Figures and Tables

**Table 1:** Comparative analysis of microscopic techniques in dermatological hair examination.

Technique	Advantages	Limitations	Resolution	Depth	Preparation	Evaluation	References
Trichoscopy	non-invasive real time, inexpensive, easy to use, and portable	limited depth, moderate resolution, and requires expertise for interpretation	at ×20 to ×160 magnifications	limited to the scalp surface; does not provide subsurface imaging	no special preparation	hair shafts, follicular openings, the epidermis surrounding the follicles, and small vessels in the skin	[4,7]
Optical Coherence Tomography	non-invasive and real time	limited penetration depth, expensive, and limited availability	between 3 and 15 μm	1–2 mm	no special preparation	hair shafts, follicular openings, small vessels in the skin, and sebaceous glands	[12,13,14]
Phototrichogram	visual documentation of hair density and growth, can assess hair cycling	dependent on image quality and requires repeated imaging	×20–×70 magnification	limited to the scalp surface; does not provide subsurface imaging	no special preparation	Hair shafts and follicular openings	[15,16]
TrichoScan	digital, software-assisted, and quantitative hair density and growth analysis	similar to phototrichogram; requires shaving and software	×20–×70 magnification	limited to the scalp surface; does not provide subsurface imaging	no special preparation	hair shafts and follicular openings	[17,18]
Scanning Electron Microscopy	extremely high resolution detailed surface topography analysis	expensive, sensitivity to sample charging and distortion	2–5 nm to 0.1–0.4 nm	50–300 µm	wash with distilled water and detergent and then dry thoroughly; requires coating with conductive materials (gold/platinum)	hair shafts and hair follicles	[19,20]
Transmission Electron Microscopy	exceptional resolution, reveals ultrastructural details, and enables intracellular imaging	expensive and inaccessible for routine use, time-consuming sample preparation, and invasive	between 0.1 and 0.2 nm	50–100 nm	mechanical plucking of hair or a biopsy; the sample in added to glutaraldehyde and osmium tetroxide, washed with buffer, dehydrated in a graded ethanol or acetone series, embedded in resin, sectioned into ultra-thin slices, and stain with uranyl acetate and lead citrate	hair shafts and hair follicles	[19,20]
Atomic Force Microscopy	atomic-scale resolution, 3D imaging, and can measure mechanical properties	slow imaging; expensive and inaccessible for routine use, non-dynamic	0.1–1 nm	10–20 µm	no special preparation	hair shafts and hair follicles	[21,22]
Reflectance Confocal Microscopy	3D high-resolution images, real-time and non-invasive	expensive, with limited depth of laser penetration	0.5–1 μm and 3–5 μm	200–250 μm	no special preparation, only oil immersion to reduce strong reflections from the hair surface	hair shafts, hair follicles, and blood vessels	[23,24,25]
Ex vivo CLSM	comparison with histopathology (since the same sample can be analyzed), sample can be re-examined	requires biopsy or hair plucking, making it invasive, non-dynamic	horizontal < 1.25 μm, vertical < 5 μm	up to 200 μm (depends on tissue type)	sample is fixed in saline or culture medium immediately after excision, can use contrast agents or fluorescent dyes for enhanced imaging	hair shafts, hair follicle, sebaceous glands, melanin distribution, cuticle, cortex, and blood vessels	[25]
High-Frequency Ultrasonography	non-invasive, safe, and accessible	limited depth of penetration	varies from 20–200 μm, depending on frequency	a 20 MHz probe can image structures 8–15 mm deep, depending on the manufacturer	no special preparation required; gel application enhances image quality	hair shafts, hair follicles, and sebaceous glands	[26,27,28]
Trichogram	simple, inexpensive, and direct hair root analysis, distinguishes anagen/telogen phases	hair plucking, discomfort, sampling errors, and time consuming	at ×100–×400	0.5 to 3 µm, limited to the scalp surface, does not provide subsurface imaging	plucking 50–100 hairs from specific area, immediate wet mount or staining	hair shafts, hair bulbs, and root sheath	[29]
Light Microscope	simple operation, good for basic structural analysis, and widely available	semi-invasive, limited depth, and sample preparation required, lower resolution than advanced tools and lower contrast for unstained samples	200 nm	0.5 to 3 µm	dry mount requires no special preparation, while wet mount involves using a potassium hydroxide solution for suspected fungal infections	hair shafts and hair follicles	[30,31]
Polarized Light Microscope	2D images, improves contrast	limited resolution, limited to birefringent structures	200 nm	0.5 to 3 µm	mounting medium for microscopy	hair shafts and hair follicles	[32]
